# Which is more troublesome, insulin or needle?

**DOI:** 10.4103/0973-3930.44082

**Published:** 2008

**Authors:** R. Menaka, U. Sowmya, P. Jegan, A. Bhattacharyya

**Affiliations:** Department of Diabetes and Endocrinology, Manipal Hospital, Bangalore, India

Dear Sir,

Initiating insulin in people with type 2 diabetes mellitus (T2DM) is a difficult task and a challenge to the clinician. There are different reasons for not accepting insulin, the most important reason being the fear of pain and needle.[1,2] There are other reasons such as insulin is seen as a last resort, “once insulin always insulin” concept, fear of low sugar and gaining weight. There is lack of data in literature as to which the patient is more worried, insulin or the injection. When injectable alternative (incretin) to insulin for treatment of T2DM were in sight a few months back, we had undertaken a project of an anonymous questionnaire survey of patients with T2DM seen in our practice taking insulin. The aim of this project was mainly to find out which is a greater barrier, insulin itself or an injection needle and to see if the decision had any bearing with hypoglycemia, duration of T2DM and duration of insulin therapy. Ethical committee approval was taken and a pilot project with ten patients was done to see clarity of the questions. We analyzed, the data of 100 consecutive patients who have completed the questionnaire. Their mean age was 46.5 years, 64 were males and 66 were graduates educated to level or above. Fifty-eight patients were on insulin for less than two years and 17 were for more than five years. A good 67 were injecting insulin by themselves. Thirty-four were ready to take an injectable alternative and 19 were undecided. Of these 34 (66% were graduates and above), fear of hypoglycemia was the major driving force in half and the rest thought new injection could be better to control diabetes. More than half of the subjects who were on insulin for more than five years and having experienced hypoglycemia within last three months preferred alternative treatment. Age did not matter in the decision making when divided into <40, 40–60 and >60 years groups, neither the sex.

Of those 47 who had said no to alternative injection, the main issue was that they were already on injection and hence, the new medicine as an injection was not acceptable. They were also more skeptical about the safety profile of the new medicine.

There were two issues which we had purposefully omitted in our survey: option for number of injections and the cost factor, knowing fully well that people would obviously prefer lesser number of injections and a cheaper alternative.

It is interesting to see in our survey that one-third of people taking insulin still would be happy to inject the newer incretin when available, the main reason being fear of hypoglycemia and prospect of better control with newer agent. Educational status had a big role to play in our cohort in the decision making. We plan to conduct a similar survey in people with T2DM who are uncontrolled with oral agents and waiting for insulin initiation to see what they would prefer, insulin or incretin.
